# IgG glycosylation dynamics in active lupus nephritis during the first year of immunosuppressive therapy

**DOI:** 10.3389/fimmu.2026.1886381

**Published:** 2026-08-18

**Authors:** Ana Cindrić, Dionysis Nikolopoulos, Natalia Sherina, Frano Vučković, Farah Tamirou, Anne-Marie Patenaude, Maja Pučić-Baković, Barbara Radovani Trbojević, Frédéric A. Houssiau, Gordan Lauc, Ioannis Parodis

**Affiliations:** 1 Genos Glycoscience Research Laboratory, Zagreb, Croatia; 2 Division of Rheumatology, Department of Medicine Solna, Centre for Molecular Medicine (CMM), Karolinska Institutet, Stockholm, Sweden; 3 Department of Gastroenterology, Dermatology, and Rheumatology, Karolinska University Hospital, Stockholm, Sweden; 4 Rheumatology Department, Cliniques Universitaires Saint-Luc, Pôle de Pathologies Rhumatismales Inflammatoires et Systémiques, Institut de Recherche Expérimentale et Clinique, Université Catholique de Louvain, Brussels, Belgium; 5 Faculty of Pharmacy and Biochemistry, University of Zagreb, Zagreb, Croatia; 6 Department of Rheumatology, Faculty of Medicine and Health, Örebro University, Örebro, Sweden

**Keywords:** disease progression, IgG glycosylation, immunosuppressive therapy, lupus nephritis, repeat kidney biopsy, Systemic lupus erythematosus

## Abstract

**Introduction:**

Lupus nephritis (LN) is a severe manifestation of systemic lupus erythematosus (SLE) characterized by immune dysregulation and kidney inflammation. Alterations in immunoglobulin G (IgG) glycosylation are known to influence immune responses and have been implicated in autoimmune diseases, including SLE and LN. This study aimed to investigate longitudinal changes in IgG glycosylation in patients with active LN, from before treatment to one year after immunosuppressive therapy initiation.

**Methods:**

Serum samples were collected from 17 patients with LN at two time points, i.e., at active LN, prior to treatment initiation (baseline), and after 12 months of therapy. IgG was purified and subjected to detailed glycosylation analysis (total and fragment-specific) by capillary gel electrophoresis (CGE). Changes in glycosylation patterns between the two time points were assessed using linear mixed effects and regression models.

**Results:**

Significant alterations in IgG glycosylation were observed between the two time points. In total IgG, decreased sialylation and increased monogalactosylated structures were prominent. Fragment-specific analyses revealed divergent dynamics: the Fc region showed increased asialylation and a higher proportion of bisecting GlcNAc-containing glycans, consistent with a pro-inflammatory effector profile, while the Fab region exhibited increased digalactosylated glycans and decreased fucosylation, a pattern not mirrored in the Fc region or total IgG glycome.

**Discussion:**

These findings indicate pro-inflammatory longitudinal shifts in IgG glycosylation during the transition of LN from an active to a treated disease state. The study underscores the relevance of IgG glycosylation in LN pathophysiology and suggests that IgG glycosylation patterns change following immunosuppressive therapy for active LN, potentially reflecting ongoing disease-related immune processes despite improvement in conventional clinical parameters.

## Graphical Abstract

**Figure:**
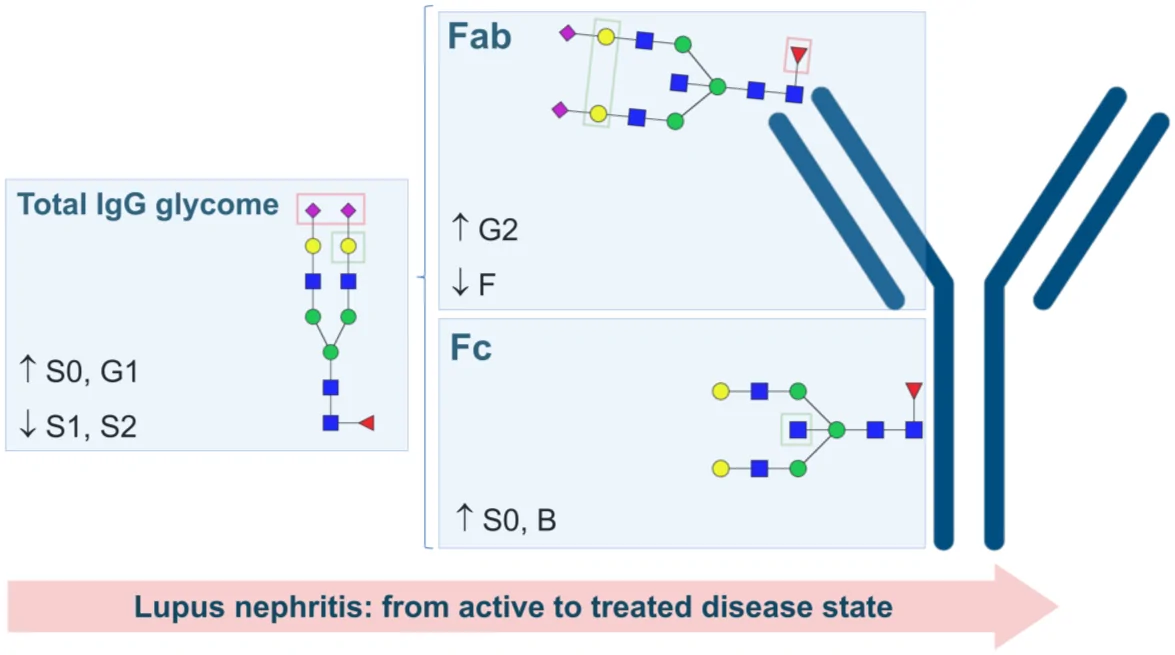

## 1 Introduction

Lupus nephritis (LN) represents one of the most severe complications of systemic lupus erythematosus (SLE), a chronic autoimmune disorder characterized by systemic inflammation and multi-organ damage. SLE disproportionately affects women, with an estimated female-to-male prevalence ratio of approximately 9:1 (1, 2). LN affects up to 60% of SLE patients during the disease course and is associated with significant morbidity and mortality (3, 4). The pathogenesis of LN involves the deposition of immune complexes in the kidneys, leading to complement activation, recruitment of inflammatory cells, and tissue damage (5). Despite advances in understanding its pathophysiology, predicting disease activity and progression is still an ongoing challenge, highlighting the need for biomarkers (6).

N-glycosylation, the enzymatic addition of carbohydrate moieties to proteins, plays an important role in modulating protein structure and function (7). Immunoglobulin G (IgG), the most abundant antibody in circulation, consists of an antigen-binding region (fragment antigen-binding, Fab) and a region that most often interacts with effector molecules and cells (fragment crystallizable, Fc). Within its Fc region, each IgG molecule contains a conserved N-glycosylation site, which has been demonstrated to significantly influence its effector functions. Specifically, changes in Fc glycosylation can modulate the ability of IgG to activate complement, engage Fc receptors, and regulate pro- and anti-inflammatory pathways (8, 9). Additionally, approximately 20% of circulating IgG molecules are glycosylated at their Fab regions, a modification that can influence antigen binding and immune complex formation (10). In autoimmune diseases, including SLE, alterations in IgG glycosylation have been implicated in disease pathogenesis and immune dysregulation, in some cases showing higher sensitivity than conventional markers (11–17).

Characterization of changes in IgG glycosylation over time could pave the way toward identification of patterns that correlate with disease progression and/or treatment response. In our previous study on SLE, we observed that the IgG glycome in SLE is significantly altered, characterized by decreased galactosylation and sialylation and increased bisecting GlcNAc, with the magnitude of these changes correlating with disease severity. To better understand the role of IgG glycosylation in SLE, evaluation of longitudinal dynamics of the IgG glycome within individual patients at different disease stages and/or following therapy is needed (12).

In the present study, we aimed to analyze IgG glycosylation profiles in LN patients at two time points: at active LN, prior to therapy initiation (baseline), and at a follow-up phase 12 months after therapy initiation, when clinical parameters had indicated improvement in most cases.

## 2 Materials and methods

### 2.1 Patient cohort

The study included 17 patients with active, biopsy-proven LN (2). Samples from the patients were collected at two time points, i.e., before initiation of immunosuppressive therapy, coinciding with the initial diagnostic kidney biopsy, and 12 months later, coinciding with a per-protocol performed repeat kidney biopsy. Patients were evaluated using standard clinical parameters for monitoring disease status.

### 2.2 IgG glycosylation analysis

IgG isolation from serum samples was performed via protein G-based affinity chromatography, followed by enzymatic N-glycan release, fluorescent labeling, and CGE-LIF detection using a well-established and robust protocol described in detail in prior reports (18, 19). Fab and Fc glycosylation were also analyzed separately using IdeS enzymatic digestion as described previously (20).

### 2.3 Statistical analysis

To minimize experimental variability in measurements, normalization and batch correction were performed on the xCGE-LIF glycan data. To enable comparability across samples and remove experimental noise, total area normalization was conducted, where the peak area of each of the 27 glycan structures (peaks [Ps]) was divided by the total electropherogram area. Normalized glycan data underwent log transformation to account for right-skewed distribution.

In addition to the 27 directly measured glycan structures, eight derived traits were computed based on these primary glycans (**Supplementary Table 1**). These derived traits reflect broader glycosylation characteristics, such as galactosylation, fucosylation, and sialylation, by summarizing patterns across multiple glycan structures. They provide insights into enzymatic activities and genetic influences on glycosylation. As these traits represent ratios and sums of original glycans, they were calculated using normalized and batch-corrected measurements that had been transformed back to proportions (via exponential transformation of batch-corrected values).

Glycan data were first transformed to a standard normal distribution (mean = 0, SD = 1) using inverse rank transformation (R package *GenABEL*), ensuring comparability of glycan effects across traits and maintaining uniform variance.

Longitudinal changes in individual and derived glycan traits of total IgG, IgG Fc, and IgG Fab were assessed using linear mixed effects models, with patient ID included as a random effect, and time included as a fixed effect. This approach allowed us to evaluate within-patient differences in glycosylation between baseline and follow-up.

The Benjamini-Hochberg procedure was applied to control the false discovery rate (FDR) at 0.05, and adjusted p-values are reported throughout. Statistical analyses and visualizations were performed in R (version 3.0.1).

## 3 Results

To characterize longitudinal IgG glycosylation dynamics in LN, we analyzed total IgG as well as the Fc and Fab regions separately across two time points in 17 patients (baseline and 12 months; patient characteristics in **Table 1**). We analyzed total IgG glycosylation and performed separate glycosylation analyses for both the Fc and Fab regions. We next assessed the change in IgG glycosylation (total, Fab, and Fc) from baseline to the 12-month follow-up (**Supplementary Table 2**).

**Table 1:** Patient characteristics.

Patient characteristic	Baseline (n=17)	Month 12 (n=17)
Age (years), mean ± SD	34.9 ± 8.7	–
Female sex, n (%)	16 (94.1)	–
European origin, n (%)	9 (52.9)	–
Non-European origin, n (%)	8 (47.1)	–
S-creatinine (mg/dL), mean (SD)	1.09 (0.54)	0.91 (0.29)
eGFR*, mean (SD)	79.9 (30.1)	89.6 (30.4)
UPCR (g/g), median (IQR)	1.65 (0.96–3.96)	0.38 (0.15–1.03)
Serum albumin (g/L), mean (SD)	29.9 (5.7)	41.9 (6.2)
SLEDAI-2K, mean (SD)	16.1 (4.7)	5.2 (3.5)
Anti-dsDNA positivity, n (%)	16 (94.1)	13 (76.5)
C3 (g/L), mean (SD)	0.57 (0.24)	0.83 (0.24)
Low C3, n (%)	16 (94.1)	10 (58.8)
C4 (g/L), mean (SD)	0.09 (0.06)	0.16 (0.06)
Low C4, n (%)	12 (70.6)	4 (23.5)
ISN/RPS class		
Class II, n (%)	0 (0)	2 (11.7)
Class III or IV, n (%)	5 (29.4)	4 (23.4)
Class III or IV + V, n (%)	10 (58.8)	6 (35.1)
Class V, n (%)	2 (11.7)	5 (29.4)
NIH activity index, median (IQR)	6 (3–8)	1 (0–3)
NIH chronicity index, median (IQR)	2 (1–2)	3 (1.5–4)
Immunosuppressive treatment**		
Intravenous low-dose cyclophosphamide, n (%)	14 (82.4)	
Intravenous rituximab, n (%)	1 (5.9)	
Oral mycophenolate mofetil, n (%)	2 (11.8)	

*Based on the CKD-EPI creatinine equation.

**Initiated at baseline, continued through follow-up.

anti-dsDNA, anti-double-stranded DNA; C3, complement component 3; C4, complement component 4; CKD-EPI, chronic kidney disease Epidemiology Collaboration; eGFR, estimated glomerular filtration rate; ISN/RPS, International Society of Nephrology/Renal Pathology Society; NIH, National Institutes of Health; SLEDAI-2K, Systemic Lupus Erythematosus Disease Activity Index 2000; UPCR, urine protein to creatinine ratio.

### 3.1 Changes in IgG glycosylation between time points

IgG glycosylation patterns demonstrated significant changes between baseline and month 12 (**Supplementary Table 2**). Notably, the glycosylation changes observed differed between the total IgG glycome, the Fc region, and the Fab region.

#### 3.1.1 Total IgG glycosylation changes over time in LN

The most common type of analysis focuses on the total IgG glycome, which contains combined information on glycosylation of both the Fc and Fab regions (9). For comparability and completeness, we first analyzed the total IgG glycome.

**Figure 1:**
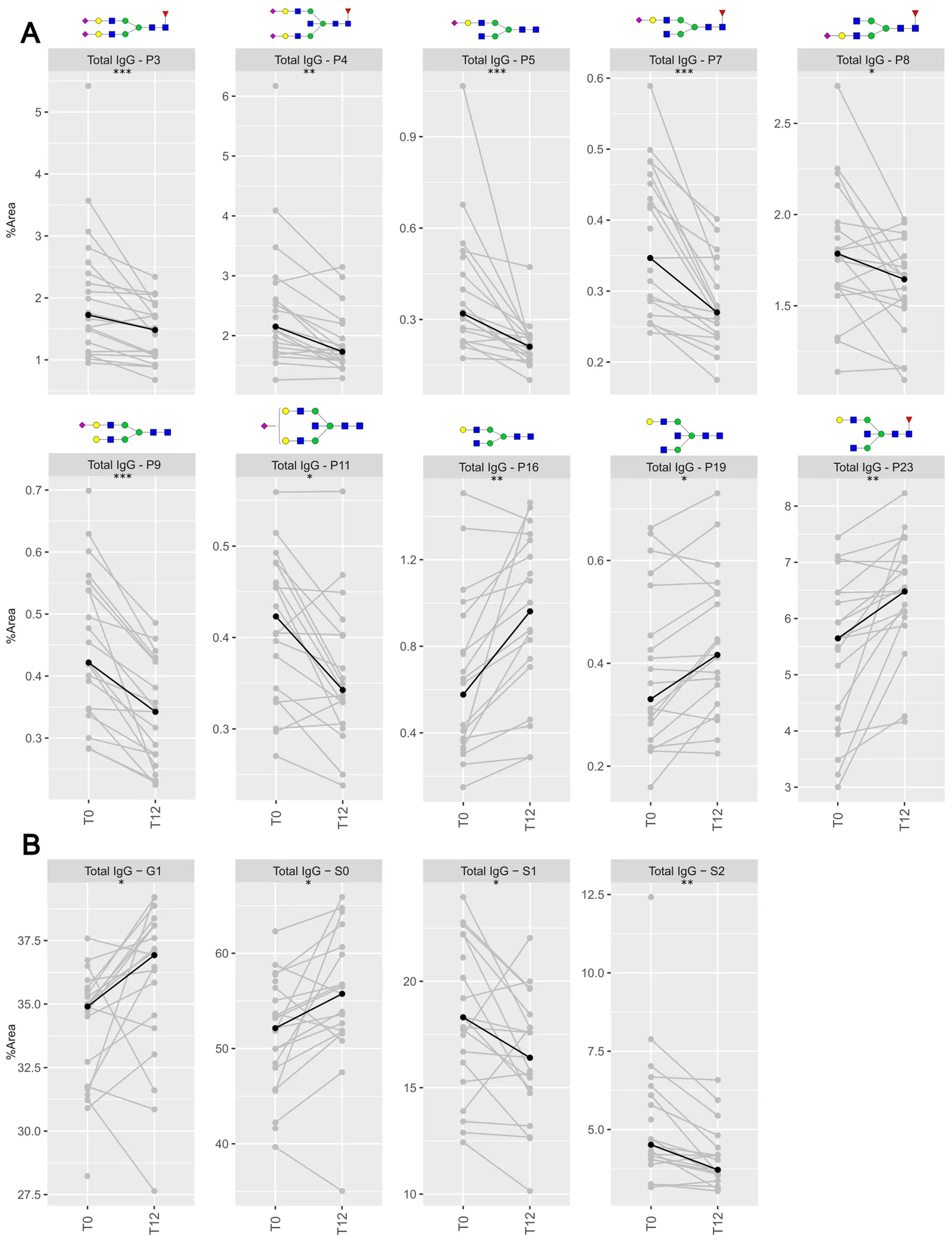
Total IgG glycan percentage changes in time in LN patients. **(A)** Individual glycan peaks with corresponding structures, **(B)** Derived glycan traits. Shown are only the glycans/traits whose percentages changed statistically significantly over time. Significance is depicted as follows: one asterisk (*) for (p<0.05), two asterisks (**) for (p<0.01), and three asterisks (***) for (p<0.001). T0 – sampled at baseline, T12 – sampled 12 months after baseline, G1 – monogalactosylated glycans, S0 – asialylated glycans, S1 – monosialylated glycans, S2 – disialylated glycans.

In the total IgG glycome, we observed a significant increase in individual glycan peaks P16 (p= 3.12E-03), P19 (p=3.58E-02), and P23 (p=1.30E-03) between time points. Each of these peaks contains mainly monogalactosylated (G1) structures. Consequently, a statistically significant increase is observed in the G1 trait (p= 3.74E-02) in the total IgG glycome (**Figure 1**). No significant change was observed in either of the other analyzed galactosylation-related traits (G0 and G2).

Sialylation changes between time points were extensive in the total IgG glycome. We noted a statistically significant decrease in both mono- (S1, p= 3.02E-02) and di-sialylated (S2, p= 1.42E-03) glycans, and an increase in glycans containing no sialic acid residues (S0, p= 4.03E-02) after therapy (**Figure 1**). This overall drop in total IgG sialylation was driven by a statistically significant decrease in as many as seven individual sialylated glycan peaks (**Figure 1**).

To assess whether the changes in total IgG glycosylation are driven by a change in the Fc portion or the Fab portion, or perhaps a combination of the two, as well as to acquire more insight into the potential functional implications, we also analyzed the two IgG fractions separately.

#### 3.1.2 IgG Fc glycosylation changes over time in LN

In revealing the specific dynamics behind the total IgG glycome changes, we first focused on the Fc region, which contains a conserved N-glycosylation site whose functional roles have been studied more extensively than those of Fab glycosylation (9).

**Figure 2:**
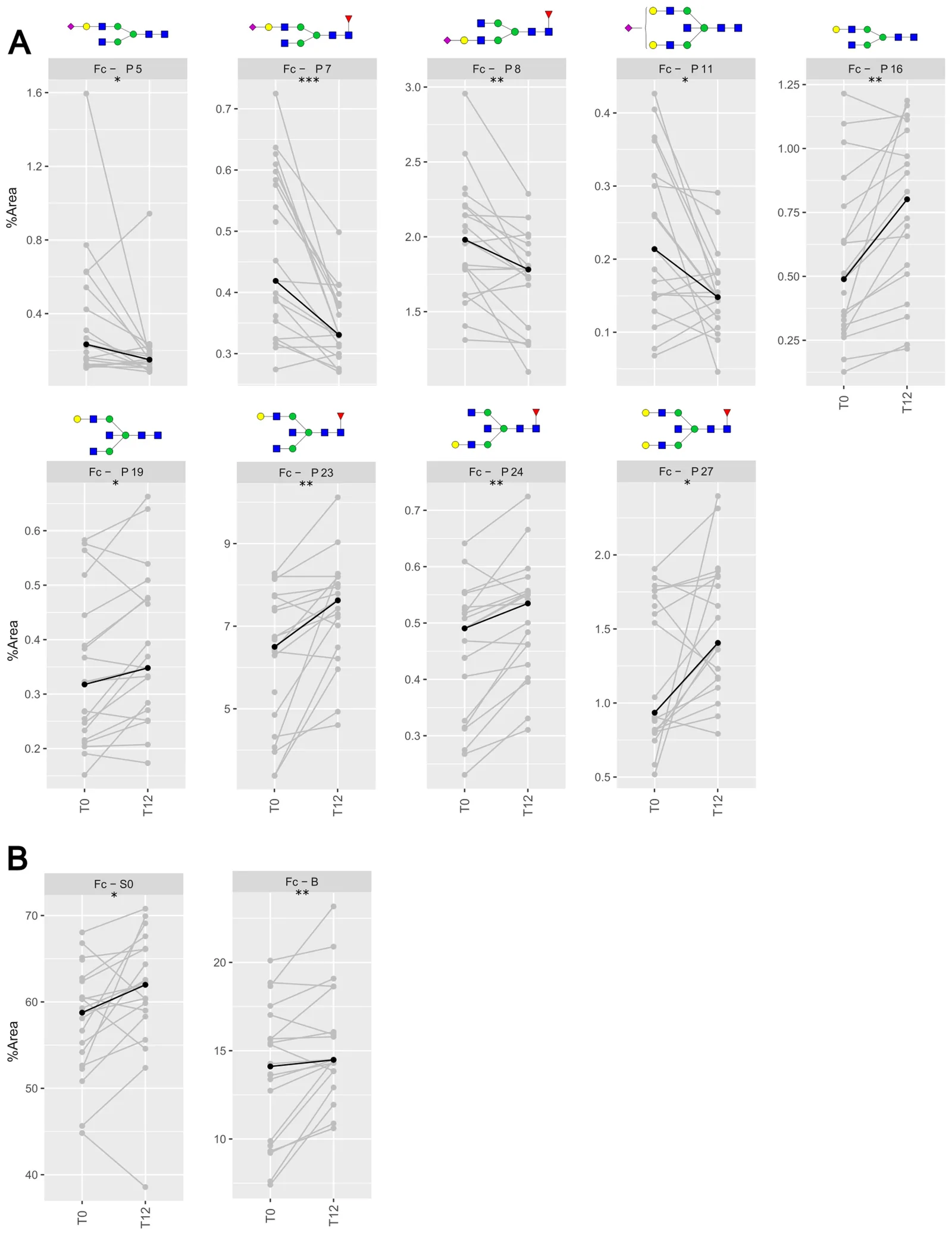
IgG Fc region glycan percentage changes in time in LN patients. **(A)** Individual glycan peaks with corresponding structures, **(B)** Derived glycan traits. Shown are only the glycans/traits whose percentages changed statistically significantly over time. Significance is depicted as follows: one asterisk (*) for (p<0.05), two asterisks (**) for (p<0.01), and three asterisks (***) for (p<0.001). T0 – sampled at baseline, T12 – sampled 12 months after baseline, S0 – asialylated glycans, B – glycans containing a bisecting GlcNAc residue.

While observing individual glycosylation traits of the total IgG glycome, similar increases in P16, P19, and P23 were observed in the Fc portion of the IgG glycome (p=5.47E-03, 2.87E-02, and 1.82E-03, respectively; **Figure 2**); such changes were not observed in the Fab region. However, even though these peaks mostly comprise monogalactosylated structures, the G1 trait was only increased in the total IgG glycome and remained unchanged in the Fc region, highlighting the importance of detailed glycome profiling.

Additionally, P24 (p=1.42E-03) and P27 (p=4.34E-02) increased over time in the Fc portion of the IgG glycome and did not change significantly in the total IgG glycome. Since both P24 and P27 are structures that contain a bisecting GlcNAc, these changes evidently accounted for the increase in B (p=1.42E-03) that was also observed in addition to the total IgG-reflecting increase in S0. This S0 increase (p=4.34E-02) was caused by a significant decrease in four individual sialylated glycans (**Figure 2**).

#### 3.1.3 IgG Fab glycosylation changes over time in LN

Fab glycosylation is usually significantly higher in autoimmune disease relative to the healthy population, remaining elevated even following B cell depletion therapy (21), and is believed to play an important role in autoimmune disease (22). As the dynamics of Fab glycosylation in LN have never been explored, we incorporated this analysis into our in-depth approach. Notably, the Fab portion of IgG showed considerably different dynamics of glycosylation changes to both Fc and total IgG.

**Figure 3:**
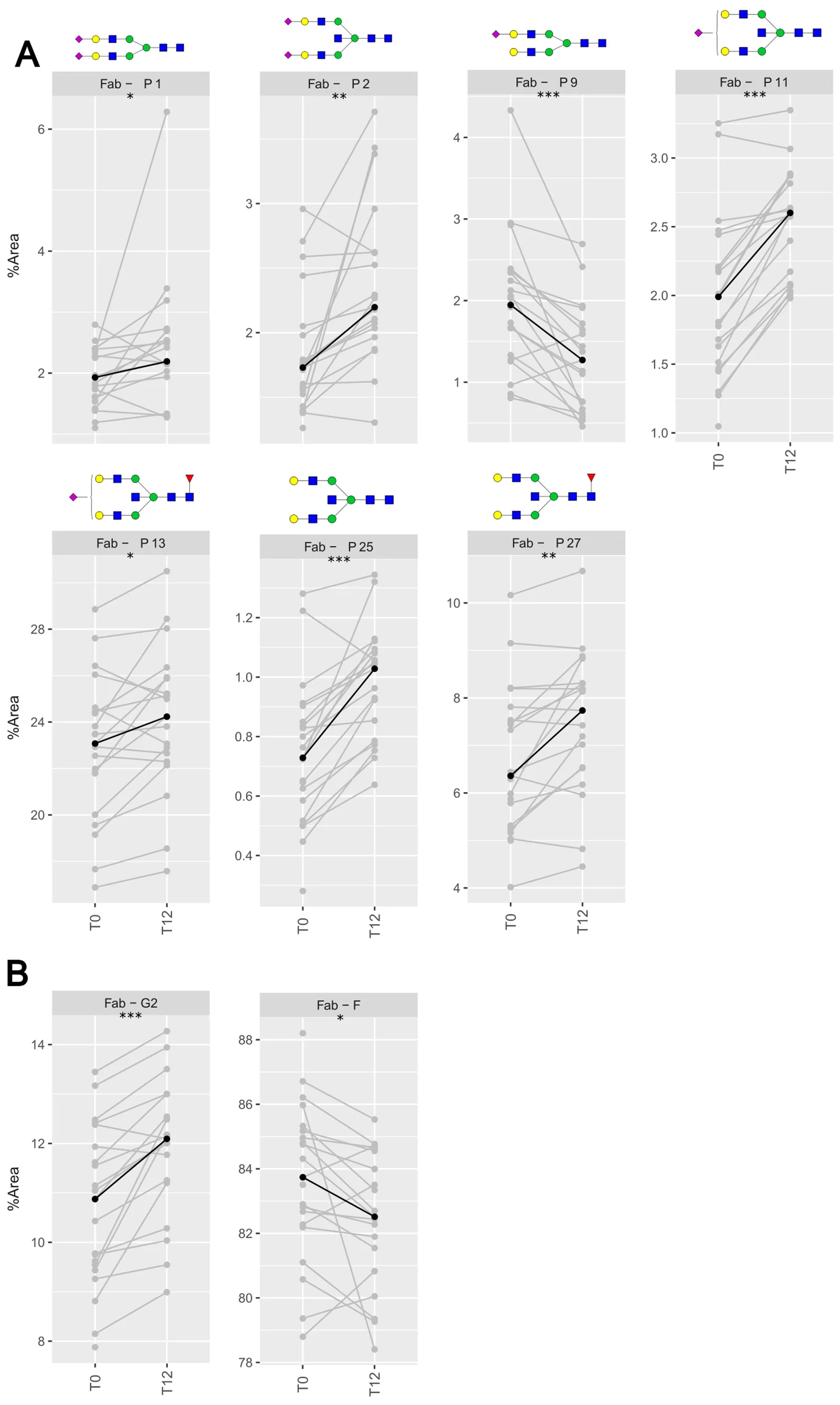
IgG Fab region glycan percentage changes in time in LN patients. **(A)** Individual glycan peaks with corresponding structures, **(B)** Derived glycan traits. Shown are only the glycans/traits whose percentages changed statistically significantly over time. Significance is depicted as follows: one asterisk (*) for (p<0.05), two asterisks (**) for (p<0.01), and three asterisks (***) for (p<0.001). T0 – sampled at baseline, T12 – sampled 12 months after baseline, G2 – digalactosylated glycans, F – fucosylated glycans.

Concretely, we observed an increase in four sialylated structures (P1, p=2.66E-02; P2, p= 1.82E-03; P11, p=7.03E-06; and P13, p=3.56E-02; **Figure 3**) after treatment, which contrastingly had a tendency to decline in the Fc region and in the total IgG glycome; as well as an increase in two digalactosylated structures, both containing a bisecting GlcNAc (P25, p=4.17E-05; and P27, p=9.26E-03). This resulted in a statistically significant increase in G2 (p=2.63E-04) and a nominal increase in S1 (p=6.39E-02, non-adjusted p=2.31E-02) on the Fab portion of IgG glycans.

P9 was the only peak whose Fab levels significantly decreased (p=3.53E-04), along with a nominal decrease in P8 (p=5.32E-02, non-adjusted p=1.77E-02) and P18 (p=5.40E-02, non-adjusted p=1.90E-02), which are all structures containing core fucose. These changes were sufficient to drive a significant decrease in F (p=4.03E-02) on Fab glycans. This pattern of change was not observed in the Fc or the total IgG glycome, which largely mirrored each other’s glycosylation patterns.

## 4 Discussion

This study investigated the hitherto unexplored longitudinal changes of IgG glycosylation in LN. By conducting separate analyses of total IgG and its Fab and Fc regions, we were able to identify glycosylation changes over the initial phase of immunosuppressive therapy for LN, transitioning from active to treated disease state. Our findings suggest that IgG glycosylation may provide unique molecular signatures of LN disease activity, which could in part reflect underlying immune dysregulation.

We observed considerable differences in IgG glycosylation patterns between baseline (active LN, prior to treatment commencement) and follow-up after one year of immunosuppressive therapy. Namely, we noted a decrease in sialylation (decreased S1 and S2, and increased S0), suggesting a pro-inflammatory immune shift during the first year after therapy initiation, as well as an increase in glycans containing one terminal galactose residue (monogalactosylation; G1) of total IgG.

Conversely, our recently published research demonstrated that total IgG glycome changes following therapy for Crohn’s disease (CD) comprised a significant increase in total IgG galactosylation and sialylation (23). Similar findings have been noted by other groups across different autoimmune conditions, as immunosuppressive therapy also resulted in a significant increase in total IgG galactosylation and sialylation in Graves’ disease (24), while multiple studies reported an increase in total IgG galactosylation following therapy in arthritis (25–28). In a longitudinal SLE cohort, decreased disease activity was associated with a complexed native IgG glycosylation profile that more closely resembled that of healthy controls compared to heightened disease activity (11). Since higher levels of total IgG galactosylation and sialylation are widely recognized as anti-inflammatory markers (9, 29), the mentioned studies’ change in glycomic pattern marks a shift toward the “healthy” state and is indicative of therapeutic efficacy on an immune biomarker level.

In contrast to these studies, we observed a decrease in IgG sialylation following treatment, evident in both total IgG glycans and the Fc region when analyzed separately. Though its role has recently been debated, diminished IgG sialylation has well-documented functional implications, exacerbating autoimmunity and impairing the anti-inflammatory capacity of IgG (9). Additionally, sialylated IgG has been demonstrated to attenuate disease development in mouse models of LN (30). This is likely because sialylated IgG exhibits enhanced binding to non-classical (type II) Fc receptors, C-type lectins, and Siglecs, thereby initiating anti-inflammatory signaling (9). Thus, the persistence of reduced sialylation on IgG despite therapeutic intervention may both reflect and contribute to ongoing pathogenic immune activity, even in patients experiencing clinical improvement, a phenomenon that has been described in prior research (31) and could explain why LN requires long treatment and maintenance therapy. A plausible cellular basis for such persistence is long-lived plasma cells, which reside in protective survival niches, are largely refractory to conventional immunosuppression and B-cell depletion (32), and secrete autoantibodies that are sufficient to drive immune complex nephritis in murine models of lupus nephritis (33).

The increase in G1, particularly in isolation from other galactosylation-related traits (G0 and G2), is a rarely reported pattern. Although immune-mediated diseases are nearly always associated with a lower overall IgG galactosylation level (15), and therapy is associated with higher overall galactosylation in longitudinal studies, to the best of our knowledge, no studies report an isolated G1 increase, highlighting the distinctiveness of LN in this regard. The underlying mechanisms remain to be explored, but selective regulation of glycosyltransferases and glycosidases, subclass-specific contributions, or distinct B-cell subset activity could play a role (9, 34).

On the Fc region, aside from the already discussed increase in S0, an increase in glycans containing bisecting GlcNAc (B) was also observed. A higher proportion of IgG glycans containing bisecting GlcNAc has consistently been observed in aging (35, 36), as well as in RA patients compared to healthy controls (37–39). The same pattern has also been observed in a study of SLE patients compared to non-SLE controls (12).

Bisecting GlcNAc has been linked to increased FcγRIIIa binding and enhanced antibody-dependent cellular cytotoxicity (ADCC), although much of this effect overlaps with the well-established proinflammatory impact of afucosylation (40). Studies that controlled for core fucosylation suggest that bisecting GlcNAc can independently enhance ADCC, though its contribution is modest compared to fucose absence, and it appears to have little effect on complement-dependent cytotoxicity (CDC) (9, 41).

Additionally, though around 10–15% of the total IgG Fc glycans contain bisecting GlcNAc, notable differences in Fc glycosylation between IgG subclasses have been reported (42). For instance, IgG1 and IgG4 have been reported to show higher levels of glycans with bisecting GlcNAc when compared to IgG2 (42). A common trait of various autoantibody responses, including in SLE, is a skewing toward the IgG4 isotype (22). It is thus possible that the differences in glycosylation between time points may partly reflect shifts in IgG subclass proportions rather than changes in glycosylation alone, as subclass distributions were not assessed in the present study.

While the Fc region of IgG predominantly governs its effector functions, antigen specificity is defined by the Fab region (43). It is well documented that the overall level of Fab glycosylation is increased in autoimmune diseases (44), as well as in disease-specific autoantibodies compared to antibodies directed against viral antigens (22), suggesting that the quantity of Fab glycosylation plays an important role in autoimmune diseases. The presence of variable domain glycosylation has also been shown to raise the activation threshold of autoreactive B cells (45), with the same research showing that the binding affinity of the Fab region can be modulated by changes in its glycosylation, with sialylation being the most prominent example (45, 46).

In this study, we observed a higher level of digalactosylated glycans (G2) and lower level of fucosylated glycans (F) on the Fab region of IgG after the initial year of immunosuppressive therapy. Compared to Fc glycosylation, the functional role of Fab glycosylation composition changes remains less well understood (10), with most qualitative research having been published relatively recently (44). As more studies focus on Fab glycosylation, a clearer perspective of its dynamics across different diseases will enable stronger associations with specific pathophysiological processes.

Taken together, our results suggest that the course of LN during the first year after therapy initiation is marked by a distinct total IgG glycosylation profile characterized by reduced sialylation and an atypical rise in G1 structures. This combination may enhance pro-inflammatory effector functions and could help explain why immunopathology sometimes persists despite apparent clinical remission (31). Future work should assess whether these glycosylation traits could serve as biomarkers of subclinical disease activity or predictors of relapse, thereby advancing their potential utility in LN patient monitoring.

Several strengths and limitations of this study should be considered when interpreting the findings. A major strength is the longitudinal design, which allowed assessment of within-patient glycosylation changes during the transition from active disease to a treated state. Such a design reduces the influence of inter-individual variability in IgG glycosylation, which is known to be substantial and influenced by genetic and environmental factors. In addition, the analysis of total IgG together with separate characterization of Fc and Fab glycosylation provided a more detailed view of IgG glycome dynamics than studies focusing solely on total IgG.

The study also has limitations. Most notably, the number of patients included in the longitudinal analysis was relatively small, which limits statistical power and may reduce the generalizability of the findings. Furthermore, although samples were collected at two clinically well-defined time points, the observational design does not allow direct attribution of the observed glycosylation changes to specific therapeutic regimens or immunological mechanisms, rendering the persistent underlying inflammation mechanism a hypothesis that remains to be mechanistically proven. Finally, neither IgG subclass distributions nor antigen-specific autoantibody glycosylation was analyzed, and therefore potential subclass or autoantibody level shifts that could contribute to the observed glycosylation patterns cannot be excluded. Replication in larger and independent cohorts will be necessary to further validate these observations and clarify their clinical relevance.

## 5 Conclusion

This study represents a longitudinal characterization of IgG glycosylation in LN, revealing distinct and fragment-specific alterations that persist despite clinical improvement under therapy. We demonstrate that the transition of LN from an active to a treated disease state is associated with a consistent decrease in IgG sialylation and a distinctive rise in monogalactosylated structures, alongside divergent dynamics in Fc and Fab glycosylation. These changes suggest that IgG glycosylation may represent a molecular signature of LN pathogenesis, reflecting immune dysregulation associated with ongoing disease-related inflammatory processes.

The distinctive glycomic trajectory we identified highlights the potential relevance of IgG glycosylation as a biomarker for disease monitoring. In addition to further studies in larger and more diverse patient cohorts, future work should also explore autoantibody-specific glycosylation, which may help identify more direct links to pathogenic mechanisms and clinical outcomes.

Nevertheless, these findings suggest that IgG glycosylation profiling may represent a promising avenue for improving our understanding of LN pathophysiology and for developing novel biomarkers that complement existing clinical measures.

## Data Availability

The original contributions presented in the study are included in the article/**Supplementary Material**. Further inquiries can be directed to the corresponding authors.
