# Supplementary material for: IgG glycosylation dynamics in active lupus nephritis during the first year of immunosuppressive therapy
Source: Front Immunol. 2026 Aug 18;17:1886381. doi: 10.3389/fimmu.2026.1886381 (PMC13529970; doi:10.3389/fimmu.2026.1886381)
Supplement: Supplementary file 1 [file SupplementaryFile1.docx]

**IgG glycosylation dynamics in active lupus nephritis during the first year of immunosuppressive therapy:**

**Supplementary Information**

| **Supplementary Table S1. Formulas for derived traits calculation.** | | |
| --- | --- | --- |
| **Trait** | **Description** | **Formula** |
| **G0** | agalactosylation | P14+P15+P18 |
| **G1** | monogalactosylation | P16+P17+P19+P20+P21+P22+P23+P24 |
| **G2** | digalactosylation | P25+P26+P27 |
| **S0** | asialylation | P14+P15+P16+P17+P18+P19+P20+P21+P22+P23+P24+P25+P26+P27 |
| **S1** | monosialylation | P5+P6+P7+P8+P9+P10+P11+P12+P13 |
| **S2** | disialylation | P1+P2+P3+P4 |
| **F** | fucosylation | P3+P4+P7+P8+P12+P13+P15+P18+P21+P22+P23+P24+P26+P27 |
| **B** | bisecting GlcNAc | P2+P4+P11+P13+P14+P18+P19+P20+P23+P24+P25+P27 |

| **Supplementary Table S2. IgG glycan changes in time in LN patients.** | | | | |
| --- | --- | --- | --- | --- |
| **Glycan** | **Time_effect** | **Time_SE** | **Time_pval** | **p.adj** |
| **Fab - P11** | 0.909 | 0.114 | 7.31E-08 | 7.03E-06 |
| **IgG - P7** | -0.948 | 0.122 | 1.34E-07 | 7.03E-06 |
| **IgG - P9** | -0.884 | 0.123 | 3.72E-07 | 1.30E-05 |
| **IgG - P5** | -1.156 | 0.180 | 1.61E-06 | 4.17E-05 |
| **Fab - P25** | 0.891 | 0.141 | 1.99E-06 | 4.17E-05 |
| **Fab - G2** | 0.664 | 0.122 | 1.50E-05 | 2.63E-04 |
| **Fc - P7** | -0.921 | 0.169 | 1.91E-05 | 2.87E-04 |
| **IgG - P3** | -0.617 | 0.118 | 2.69E-05 | 3.53E-04 |
| **Fab - P9** | -0.884 | 0.169 | 3.03E-05 | 3.53E-04 |
| **IgG - P23** | 0.780 | 0.169 | 1.23E-04 | 1.30E-03 |
| **IgG - S2** | -0.643 | 0.144 | 1.63E-04 | 1.42E-03 |
| **Fc - P24** | 0.539 | 0.118 | 1.75E-04 | 1.42E-03 |
| **Fc - B** | 0.509 | 0.113 | 1.76E-04 | 1.42E-03 |
| **Fab - P2** | 0.872 | 0.206 | 2.58E-04 | 1.82E-03 |
| **Fc - P23** | 0.702 | 0.162 | 2.61E-04 | 1.82E-03 |
| **Fc - P8** | -0.690 | 0.163 | 2.98E-04 | 1.95E-03 |
| **IgG - P4** | -0.560 | 0.138 | 4.42E-04 | 2.73E-03 |
| **IgG - P16** | 0.631 | 0.158 | 5.35E-04 | 3.12E-03 |
| **Fc - P16** | 0.588 | 0.157 | 9.89E-04 | 5.47E-03 |
| **Fab - P27** | 0.427 | 0.122 | 1.76E-03 | 9.26E-03 |
| **IgG - P11** | -0.601 | 0.176 | 2.28E-03 | 1.14E-02 |
| **Fc - P5** | -0.708 | 0.227 | 4.64E-03 | 2.22E-02 |
| **Fc - P11** | -0.741 | 0.246 | 5.77E-03 | 2.63E-02 |
| **Fab - P1** | 0.645 | 0.219 | 6.21E-03 | 2.66E-02 |
| **IgG - P8** | -0.571 | 0.192 | 6.33E-03 | 2.66E-02 |
| **Fc - P19** | 0.407 | 0.138 | 7.11E-03 | 2.87E-02 |
| **IgG - S1** | -0.556 | 0.193 | 7.77E-03 | 3.02E-02 |
| **Fab - P13** | 0.359 | 0.127 | 9.49E-03 | 3.56E-02 |
| **IgG - P19** | 0.387 | 0.138 | 9.88E-03 | 3.58E-02 |
| **IgG - G1** | 0.683 | 0.249 | 1.07E-02 | 3.74E-02 |
| **IgG - S0** | 0.571 | 0.210 | 1.21E-02 | 4.03E-02 |
| **Fab - F** | -0.542 | 0.203 | 1.23E-02 | 4.03E-02 |
| **Fc - P27** | 0.723 | 0.272 | 1.37E-02 | 4.34E-02 |
| **Fc - S0** | 0.549 | 0.206 | 1.40E-02 | 4.34E-02 |
| **Fab - P8** | -0.708 | 0.282 | 1.77E-02 | 5.32E-02 |
| **Fc - P10** | 0.603 | 0.241 | 1.88E-02 | 5.40E-02 |
| **Fab - P18** | -0.568 | 0.226 | 1.90E-02 | 5.40E-02 |
| **Fc - S1** | -0.468 | 0.193 | 2.31E-02 | 6.39E-02 |
| **IgG - P27** | 0.581 | 0.245 | 2.68E-02 | 7.22E-02 |
| **IgG - P13** | -0.272 | 0.119 | 2.99E-02 | 7.86E-02 |
| **IgG - P25** | 0.325 | 0.143 | 3.46E-02 | 8.86E-02 |
| **Fc - P3** | -0.437 | 0.206 | 4.14E-02 | 1.03E-01 |
| **Fab - S1** | 0.449 | 0.218 | 4.80E-02 | 1.17E-01 |
| **IgG - P17** | 0.351 | 0.182 | 6.19E-02 | 1.46E-01 |
| **Fab - P23** | -0.427 | 0.218 | 6.31E-02 | 1.46E-01 |
| **Fab - P6** | -0.565 | 0.299 | 6.39E-02 | 1.46E-01 |
| **Fab - P4** | -0.321 | 0.171 | 6.77E-02 | 1.49E-01 |
| **Fab - G0** | -0.378 | 0.199 | 6.81E-02 | 1.49E-01 |
| **IgG - G2** | 0.399 | 0.213 | 7.29E-02 | 1.56E-01 |
| **IgG - P6** | 0.330 | 0.179 | 7.56E-02 | 1.59E-01 |
| **Fc - P2** | 0.501 | 0.282 | 9.15E-02 | 1.86E-01 |
| **Fab - B** | 0.220 | 0.127 | 9.27E-02 | 1.86E-01 |
| **IgG - P22** | 0.301 | 0.173 | 9.38E-02 | 1.86E-01 |
| **Fab - P19** | -0.417 | 0.243 | 1.02E-01 | 1.98E-01 |
| **Fc - G2** | 0.354 | 0.211 | 1.06E-01 | 2.01E-01 |
| **IgG - P26** | 0.347 | 0.208 | 1.07E-01 | 2.01E-01 |
| **Fc - P26** | 0.347 | 0.210 | 1.11E-01 | 2.03E-01 |
| **Fab - P14** | 0.317 | 0.189 | 1.13E-01 | 2.03E-01 |
| **Fc - P1** | 0.440 | 0.271 | 1.14E-01 | 2.03E-01 |
| **Fab - G1** | -0.324 | 0.197 | 1.16E-01 | 2.03E-01 |
| **Fc - P9** | -0.283 | 0.183 | 1.31E-01 | 2.26E-01 |
| **Fab - P20** | 0.267 | 0.174 | 1.34E-01 | 2.26E-01 |
| **Fc - G1** | 0.402 | 0.262 | 1.36E-01 | 2.26E-01 |
| **Fc - P20** | -0.442 | 0.294 | 1.43E-01 | 2.34E-01 |
| **Fab - P3** | -0.216 | 0.151 | 1.62E-01 | 2.61E-01 |
| **IgG - P14** | -0.295 | 0.214 | 1.77E-01 | 2.77E-01 |
| **Fc - P12** | -0.307 | 0.222 | 1.77E-01 | 2.77E-01 |
| **Fc - P6** | 0.214 | 0.158 | 1.83E-01 | 2.82E-01 |
| **Fab - P26** | 0.221 | 0.164 | 1.87E-01 | 2.84E-01 |
| **IgG - P12** | -0.271 | 0.212 | 2.08E-01 | 3.11E-01 |
| **Fc - P21** | -0.327 | 0.262 | 2.19E-01 | 3.24E-01 |
| **IgG - P10** | 0.241 | 0.196 | 2.25E-01 | 3.29E-01 |
| **Fc - P25** | 0.310 | 0.269 | 2.55E-01 | 3.66E-01 |
| **Fab - P7** | 0.305 | 0.268 | 2.58E-01 | 3.66E-01 |
| **Fc - P15** | -0.265 | 0.241 | 2.78E-01 | 3.89E-01 |
| **Fc - P18** | 0.164 | 0.150 | 2.82E-01 | 3.90E-01 |
| **Fab - S2** | -0.185 | 0.179 | 3.05E-01 | 4.15E-01 |
| **IgG - G0** | -0.241 | 0.242 | 3.26E-01 | 4.39E-01 |
| **IgG - P20** | -0.280 | 0.285 | 3.34E-01 | 4.44E-01 |
| **Fab - P24** | -0.185 | 0.191 | 3.43E-01 | 4.47E-01 |
| **IgG - P15** | -0.243 | 0.254 | 3.45E-01 | 4.47E-01 |
| **IgG - P2** | 0.189 | 0.200 | 3.57E-01 | 4.54E-01 |
| **Fc - P17** | 0.179 | 0.194 | 3.59E-01 | 4.54E-01 |
| **Fc - G0** | -0.212 | 0.235 | 3.71E-01 | 4.56E-01 |
| **IgG - B** | 0.120 | 0.133 | 3.72E-01 | 4.56E-01 |
| **Fc - S2** | -0.238 | 0.267 | 3.74E-01 | 4.56E-01 |
| **IgG - P18** | 0.139 | 0.159 | 3.87E-01 | 4.67E-01 |
| **Fab - P5** | -0.224 | 0.287 | 4.37E-01 | 5.21E-01 |
| **Fab - S0** | 0.138 | 0.183 | 4.52E-01 | 5.33E-01 |
| **Fc - P22** | 0.141 | 0.188 | 4.59E-01 | 5.35E-01 |
| **IgG - P1** | 0.131 | 0.205 | 5.26E-01 | 6.07E-01 |
| **Fab - P17** | 0.163 | 0.304 | 5.95E-01 | 6.79E-01 |
| **Fc - F** | -0.107 | 0.205 | 6.01E-01 | 6.79E-01 |
| **IgG - F** | -0.074 | 0.168 | 6.60E-01 | 7.37E-01 |
| **Fc - P4** | -0.111 | 0.263 | 6.75E-01 | 7.37E-01 |
| **Fc - P14** | -0.103 | 0.247 | 6.76E-01 | 7.37E-01 |
| **Fab - P21** | 0.128 | 0.311 | 6.81E-01 | 7.37E-01 |
| **Fab - P22** | -0.117 | 0.292 | 6.89E-01 | 7.37E-01 |
| **Fab - P15** | 0.084 | 0.215 | 6.95E-01 | 7.37E-01 |
| **IgG - P21** | -0.091 | 0.255 | 7.20E-01 | 7.57E-01 |
| **Fab - P10** | -0.061 | 0.183 | 7.43E-01 | 7.72E-01 |
| **Fab - P16** | 0.063 | 0.311 | 8.39E-01 | 8.53E-01 |
| **Fc - P13** | 0.058 | 0.291 | 8.44E-01 | 8.53E-01 |
| **IgG - P24** | 0.027 | 0.138 | 8.45E-01 | 8.53E-01 |
| **Fab - P12** | -0.001 | 0.132 | 9.93E-01 | 9.93E-01 |
